# Toward solving the global green–green dilemma between wind energy production and bat conservation

**DOI:** 10.1093/biosci/biae023

**Published:** 2024-04-10

**Authors:** Christian C Voigt, Enrico Bernard, Joe Chun-Chia Huang, Winifred F Frick, Christian Kerbiriou, Kate MacEwan, Fiona Mathews, Armando Rodríguez-Durán, Carolin Scholz, Paul W Webala, Justin Welbergen, Michael Whitby

**Affiliations:** Leibniz Institute for Zoo and Wildlife Research, Berlin, Germany; Laboratório de Ciência Aplicada a Conservação da Biodiversidade, Universidade Federal de Pernambuco, Recife, Brazil; Department of Life Science at the National Taiwan Normal University, Taipei City, Taiwan; Bat Conservation International, Austin, Texas, United States; Centre d'Ecologie et des Sciences de la Conservation at the Muséum national d'Histoire naturelle and the Centre National de la Recherche Scientifique at Sorbonne Université Station Marine, in Concarneau, France; Western EcoSystems Technology, in Cheyenne, Wyoming, United States; School of Life Sciences at the University of Sussex, Falmer, England, United Kingdom; Universidad Interamericana, in Bayamón, Puerto Rico; Leibniz Institute for Zoo and Wildlife Research, Berlin, Germany; Department of Forestry and Wildlife Management at Maasai Mara University, Narok, Kenya; The Hawkesbury Institute for the Environment at Western Sydney University, Richmond, Victoria, Australia; Bat Conservation International, Austin, Texas, United States

**Keywords:** bats, biodiversity crisis, climate change, renewable energies, wind turbines

## Abstract

Wind energy production is growing rapidly worldwide in an effort to reduce greenhouse gas emissions. However, wind energy production is not environmentally neutral. Negative impacts on volant animals, such as bats, include fatalities at turbines and habitat loss due to land-use change and displacement. Siting turbines away from ecologically sensitive areas and implementing measures to reduce fatalities are critical to protecting bat populations. Restricting turbine operations during periods of high bat activity is the most effective form of mitigation currently available to reduce fatalities. Compensating for habitat loss and offsetting mortality are not often practiced, because meaningful offsets are lacking. Legal frameworks to prevent or mitigate the negative impacts of wind energy on bats are absent in most countries, especially in emerging markets. Therefore, governments and lending institutions are key in reconciling wind energy production with biodiversity goals by requiring sufficient environmental standards for wind energy projects.

Wind power generation has become a pillar of renewable energy generation worldwide in the fight against global warming (GWEC 2023). However, wind energy generation is not environmentally neutral; that is, wind turbines can have negative impacts on local habitats and the organisms that live in them (Saidur et al. 2011, Gibson et al. 2017). For example, the construction of wind turbine facilities may fragment and degrade habitats, making them unsuitable for breeding, foraging, commuting, and migration (Shaffer and Buhl 2016, Reusch et al. 2022). Furthermore, large numbers of volant vertebrates, such as raptors and bats (Thaxter et al. 2017), are killed by wind turbines. With bats accounting for the majority of vertebrate fatalities at wind turbines, global wind energy production is the leading cause of multiple mortality events in bats (O'Shea et al. 2016). Bats are an essential component of mammalian diversity (Simmons and Cirranello 2024). They are key parts of ecological communities and are important bioindicators in almost all ecosystems (Jones et al. 2009). They also provide ecosystem services to agriculture and forestry (Kunz et al. 2011). However, more than one-third of bat species are threatened or data deficient, according to the International Union for Conservation of Nature's (IUCN) standards, and about half show negative population trends (Frick et al. 2020). Reducing the negative impacts of wind energy on bats is an important conservation objective. Over the past two decades, the body of literature on the impacts of wind energy on bats and effective mitigation measures has grown considerably, although we still lack data for many regions of the world, particularly for countries with emerging markets (figure 1). In the present article, we review the interactions of bats with wind turbines, discuss the limitations of our understanding on a global scale, and highlight effective measures to mitigate this green–green dilemma.

**Figure 1.**
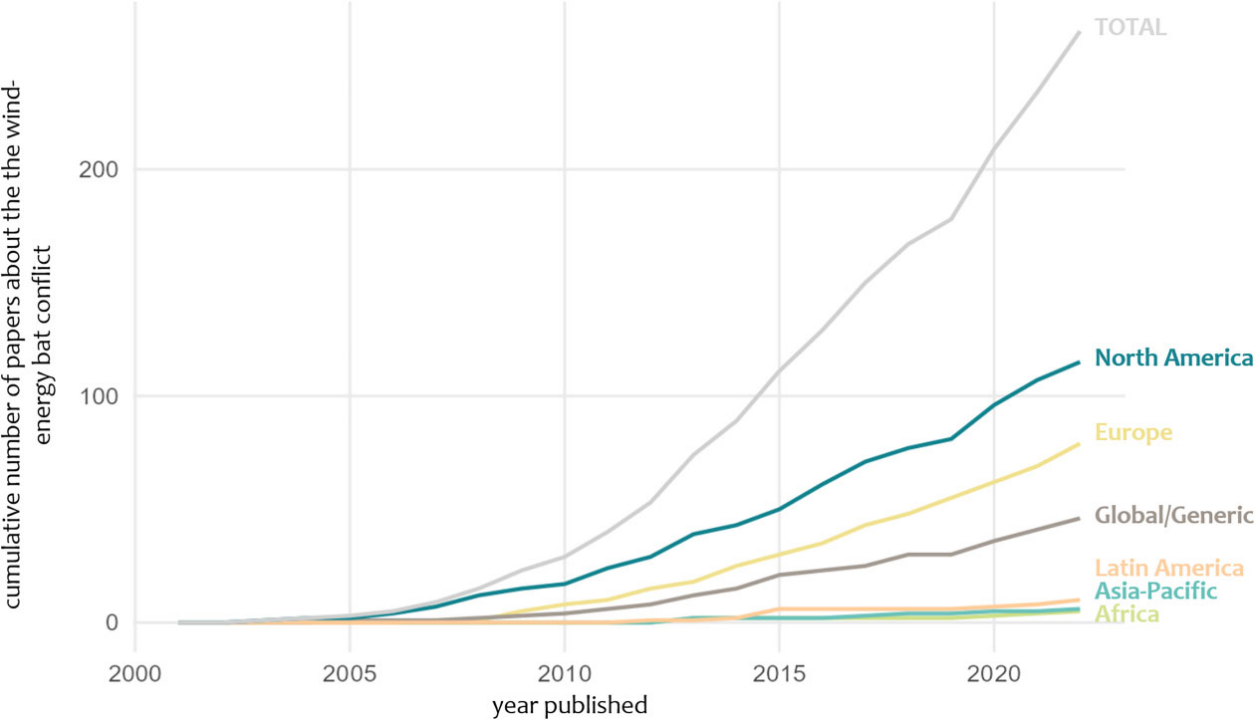
The cumulative number of papers on the green–green dilemma between wind energy production and bat conservation in peer-reviewed journals. The cumulative numbers were extracted by a literature search (see the supplemental material). The results were assigned to five major regions—North America, Latin America, Europe, Africa, and Asia-Pacific (following Global Wind Energy Council 2023)—and a sixth category covering global or generic aspects, such as modeling papers or review papers spanning more than one region.

## Global wind energy expansion

**Figure 2.**
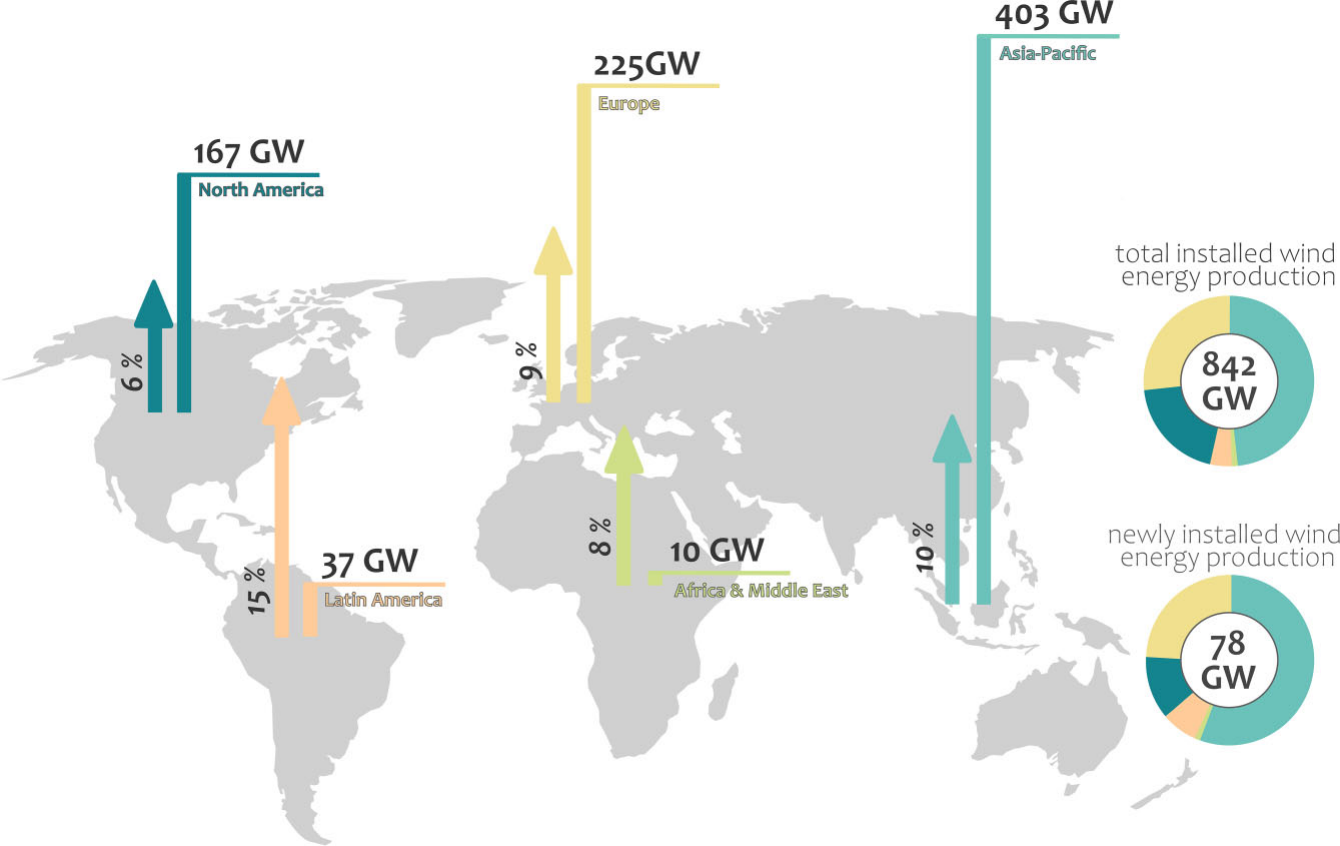
The total installed wind energy production and the percentage increase in wind energy production in 2022 relative to the total installed wind energy production for the five major regions defined by the Global Wind Energy Council (2023): North America, Latin America, Europe, Africa, and Asia-Pacific.

Worldwide, wind energy production is growing by 10%–20% per year (IRENA 2022). By the end of 2022, the total installed wind power capacity was 841.9 gigawatts (GW) for onshore and 64.3 GW for offshore (GWEC 2023). In 2022, newly installed wind power capacity equaled 77.6 GW (GWEC 2023). The largest increases are occurring in the Asia-Pacific, the world's largest wind market, with China making the largest contribution (87%; figure 2; GWEC 2023). Emerging markets in the tropical belt and the Southern Hemisphere contribute significantly to the regional increase in installed wind power, particularly Brazil, India, Chile, Vietnam, Colombia, and Mozambique (GWEC 2023). Many of these emerging markets overlap global biodiversity hotspots (Mittermeier et al. 2011, Alves et al. 2018, Frick et al. 2020). Therefore, major land-use changes, such as those associated with the expansion of wind energy production, pose a high risk to the conservation of volant wildlife, including bats.

Box 1. **Surveying bats at wind energy facilities.**

**Figure 3.**
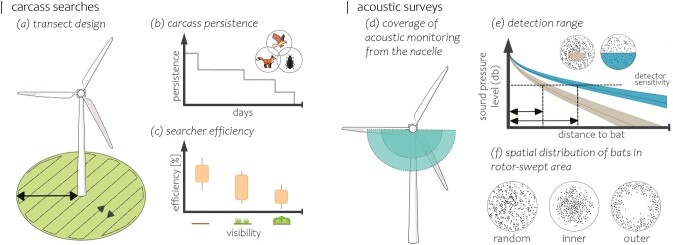
Schematic illustration of monitoring the risk of bat mortality based on carcasses searches (a-c) and acoustic monitoring (d-f).

The risk of bat mortality at wind turbines can be monitored directly and indirectly: Bat carcasses can be counted under wind turbines by searching a predefined area around the tower (figure 3a). Search campaigns need to be carried out regularly and systematically. Importantly, complementary experiments are needed to quantify the disappearance of carcasses because of natural decay, scavengers and agricultural practices (figure 3b), and the efficiency of searchers (figure 3c) in areas of varying visibility. The results of these experiments are used to estimate mortality rates (Huso et al. 2016, Dalthorp et al. 2018). A good practice manual was recently published providing guidance on postconstruction fatality monitoring (IFC 2023).

Bat activity around wind turbines can also be estimated, typically using acoustic monitoring, either from the ground or from detectors mounted on the nacelle or on the tower (figure 3d). The detection ranges of ultrasonic detectors depend on the ultrasonic frequencies and sound pressure levels of echolocating bats (Voigt et al. 2021). Figure 3e shows the attenuation of echolocation calls for a species with high ultrasonic frequencies (gray; e.g., around 45 kilohertz) and low frequency echolocation calls (blue; around 20–25 kilohertz; Voigt et al. 2021). Also, varying spatial distributions of bat passes (the dots in figure 3e and 3f) within the rotor swept area (the circle in figure 3e and 3f) may affect the power of acoustic monitoring to predict mortality rates (figure 3f; Voigt et al. 2022b). Although there are benefits to monitoring bat acoustic activity in the rotor-swept area, the relationships between activity and collision risk can be complex and nonlinear (Mathews et al. 2016), so the effectiveness of acoustic monitoring should be validated with carcass-based fatality estimates. This is particularly true given the recent trend toward larger wind turbines, which reduces the relative coverage of the rotor swept area by ultrasonic detectors. Carcass and acoustic surveys have been conducted primarily at wind turbines in North America and Europe. Therefore, we lack important information on how best to conduct carcass searches and estimate mortality rates at wind turbines in regions with more diverse bat faunas and those with nonecholocating bat species. Radar may be useful for monitoring nonecholocating species or as an alternative to acoustic monitoring, but has not been as widely used, in part because of its much higher cost. On a global scale, furthermore, studies are needed to measure the relationship between acoustic activity and mortality, and to identify when the predictive power of acoustic monitoring is limited, for example, because of variations in turbine size or characteristics of local bat assemblages.

## Impact of wind energy production on bats

Wind energy production can affect bats in three different ways. Bats can collide with the rotating blades, they can lose important habitats and they can get displaced by the operation of the turbines.

### Direct mortality

Bat carcasses have been found under wind turbines since the beginning of wind energy production (Kunz et al. 2007, Arnett et al. 2008, Rydell et al. 2010). Over the past decade, researchers have improved methods for estimating bat mortality (box 1), and the rates range from a few individuals to more than 100 bats per turbine per year (Hein et al. 2013, AWWI 2020, Voigt et al. 2022a). In North America and Central Europe, the fatality rates are approximately 12 and 14 bats per turbine per year, respectively (AWWI 2020, Voigt et al. 2022a), which translates to 6–7 bat fatalities per year per megawatt (MW) of installed capacity. In South Africa, estimated fatality rates average three bats per MW produced per year (Aronson 2022). In Latin America, wind turbines cause highly variable bat mortality rates, ranging from 2 to 57 bats per MW per year (Barros 2019, Agudelo et al. 2021). The estimated annual losses of bats killed by wind turbines are about 30,000 bats per year in the United Kingdom, about 50,000 bats per year in Canada, more than 200,000 bats per year in Germany, and more than 500,000 bats per year in the United States (Mathews et al. 2016, Zimmerling and Francis 2016, Voigt et al. 2019, AWWI 2020). Such estimates are not readily available for other regions. Fatalities may be even higher in the tropics, where bat abundance and species richness are highest (Alves et al. 2018, Frick et al. 2020).

The species at high risk of collision are typically aerial insectivores, particularly the functional guilds of open-space and edge-space foraging bats (table 1; Rydell et al. 2010), which forage and commute at the level of the turbine blades (Roeleke et al. 2016, Roemer et al. 2017, O'Mara et al. 2019, McCracken et al. 2021, Reusch et al. 2023). In temperate areas, mortality varies seasonally, typically increasing in late summer as the bats leave their maternity roosts after the reproductive season (Cryan 2003, Kruszynski et al. 2022). However, this pattern may differ in regions without strong seasonal climates, especially those closer to the equator (Aronson 2022). For migratory species, the impact of wind turbines can extend over large areas or even continents (Voigt et al. 2012, Baerwald et al. 2014, Lehnert et al. 2014).

**Table 1.** Potential impacts of wind turbines on bats varies between taxonomic groups and functional guilds.

	**Functional guild**		**Threat**		
**Taxon**	**Habitat**	**Foraging style,example taxa**	**Fatalities**	**Habitat loss**	**Displacement**
Yangochiroptera	Open space	Aerial insectivores, e.g., some Vespertilionidae, all Molossidae, some Mormoopidae and Emballonuridae	High^c–i, l,m, r,u–y,ab,ac,ad^	Medium^n^	High^n,o,s,t,z,aa,ae^
	Edge space	Aerial insectivores, e.g., some Vespertilionidae, some Emballonuridae	High^j,l,m,v–y,ac,ad^	Unknown	High^r,z,aa^
	Edge space	Trawling, e.g., some Vespertilionidae, Noctilionidae	Low to medium^c,w,ad^	*Low risk*	Unknown
	Narrow space	Active gleaning, e.g., some Phyllostomidae	Low to medium risk^a^, ^x,y,ab,ad^	*High risk*	Unknown
	Narrow space	Passive gleaning, e.g., some Vespertilionidae, Nycteridae	Low risk^c–i,l,m,x,ac^	*High risk*	High risk ^p,q, r,t,z,aa,ae,af^
Yinpterochiroptera	Open and edge space	Fruit-eating pteropodids	High risk^ac^	*Low to medium risk*	Unknown
	Edge and narrow space	Nectar-feeding pteropodids	Unknown	*High risk*	Unknown
	Edge space	Aerial insectivores, i.e., Craseonycteridae	Unknown	Unknown	Unknown
	Narrow space	Flutter-detecting bats, e.g., Rhinolophidae	Low risk^a,b,c^	Unknown	Unknown
	Narrow space	Active gleaning, i.e., Megadermatidae	*Low risk*	Unknown	Unknown

*Note:* Echolocating bat species were assigned to functional groups according to Denzinger and Schnitzler (2013). Threat assignments are based on a literature search. Notably, potential impacts of wind turbines may vary among species within functional guilds. Threat assessments are shown in italics when inferred from the general biology of bats; for example, forest-dependent bats were considered to suffer from habitat loss because of the logging involved when turbines are constructed in forests. The taxonomic division of Yangochiroptera and Yimpterchiroptera follows Teeling et al. 2005. ^a^Likely high risk when wind turbines are placed next to large colonies (e.g., cave-roosting species).  ^b^Likely higher risk when wind turbines are placed at commuting routes for some large species (e.g., *Hipposideros armiger*) that usually fly above canopy during commuting. *Sources:*  ^c^Rydell et al. (2010). ^d^Rodrigues et al. 2015. ^e^Voigt et al. 2015. ^f^Arnett and Baerwald 2013. ^g^Arnett et al. 2008. ^h^Baerwald et al. 2014. ^i^Hein et al. 2013. ^j^Kruszynski et al. 2022. ^k^Lehnert et al. 2014. ^l^Măntoiu et al. 2020. ^m^Roemer et al. 2017. ^n^Reusch et al. 2022. ^o^Reusch et al. 2023. ^p^Ellerbrok et al. 2022. ^q^Ellerbrok et al. 2024. ^r^Cryan et al. 2014. ^s^Millon et al. 2018. ^t^Gaultier et al. 2023. ^u^Roeleke et al. 2016. ^v^Voigt et al. 2012. ^w^Voigt et al. 2022b. ^x^Cabrera-Cruz et al. 2020. ^y^Rodríguez-Durán and Feliciano-Robles 2015. ^z^Leroux et al. 2022. ^aa^Barré et al. 2018. ^ab^Barros et al. 2019. ^ac^Aronson 2022. ^ad^Agudelo et al. 2021. ^ae^Barré et al. 2018. ^af^Mckay et al. 2023.

Wind energy installations can cause rapid population declines, potentially placing species at risk of regional extinction. For example, population models based on demographic data and wind-turbine-related fatalities of hoary bats (*Lasiurus cinereus*) predict population declines for this species in North America (Frick et al. 2017, Friedenberg and Frick 2021). Population declines have been reported in central Europe for species with high collision risk, such as the noctule bat *Nyctalus noctula* (EUROBATS 2018, Bas et al. 2020, Printz et al. 2021). In contrast, European species with no or low risk of collision appear to have stable to increasing population trends, because their populations are gradually recovering from historical population lows caused by the unregulated pesticide use in the midtwentieth century (Van der Meij et al. 2015). Given that other anthropogenic stressors did not emerge concurrently with the boom in wind energy production and that population declines are restricted to species at high risk of collision, it is likely that these declines are related to the expansion of wind energy production.

In emerging markets, bats at high risk of collision may belong to other functional guilds—for example, understory frugivores in the Neotropics (Barros et al. 2015, Rodríguez-Durán and Feliciano-Robles 2015, Cabrera-Cruz et al. 2020), especially when the turbines are located near large roosts, such as hot caves in the Caribbean (Ladle et al. 2012). In the Old World, pteropodids are also vulnerable to collisions with wind turbines (Aronson 2022).

Some bats are attracted to wind turbines (Cryan et al. 2014, Lintott et al. 2016, Richardson et al. 2021, Reusch et al. 2023), which could increase the magnitude of population reduction of vulnerable bat species. Clearing forests for wind turbines creates habitat for open- and edge-space-foraging bats, resulting in increased activities of these species at high risk of collision near wind turbines in forested areas (Ellerbrok et al. 2023). Attraction appears to vary between taxa and may depend on season and local habitat (Jameson and Willis 2014, Leroux et al. 2022), but besides habitat conversion the underlying drivers of attraction are currently unknown (Guest et al. 2022). Reusch and colleagues observed both attraction and avoidance of individual bats of the same species toward wind turbines (Reusch et al. 2022, 2023). Possibly, local bats avoided wind turbines, whereas conspecifics from other regions were attracted to wind turbines—for example, in search of roosts (Cryan et al. 2014). Flight height also appears to influence vulnerability to collisions with wind turbines (McCracken et al. 2008, Cryan et al. 2014, Roeleke et al. 2016, Roemer et al. 2017, Reusch et al. 2023). Both flight altitude and movement status (migratory or commuting, as in the case of fruit-eating phyllostomids and pteropodids) may predispose bats to collide with the spinning blades of wind turbines.

### Habitat loss

Over the past decades, wind turbines have been sited mostly in open or semiopen landscapes because of the high efficiency of wind energy production, improved infrastructure accessibility, legal frameworks, or dual land-use benefits (Hise et al. 2022, Weber et al. 2023). However, the current high demand for wind energy is putting pressure on land in more vegetated and topographically complex landscapes, often with relatively low potential for wind energy production (Schöll and Nopp-Mayr 2021). Forests are sometimes cleared before turbines are installed, which can lead to the destruction of priority areas for biodiversity, as in Brazil, for example (Neri et al. 2019). Alternatively, smaller patches within forests can be cleared and turbines installed in keyholes. The size of such cleared patches is primarily determined by the spatial requirements for the installation and maintenance of wind turbines (Quentin and Tucci 2022). According to Denholm and colleagues (2009), the area permanently converted for wind turbines on forested sites averages 0.36 hectares [ha] per MW (direct impact area; with a standard deviation of 0.22), and the area temporarily converted 1.14 ha per MW (indirect impact area; with a standard deviation of 1.2). Therefore, a 2.5 MW wind turbine will result in permanent conversion of 0.9 ha and temporary conversion of 2.78 ha of forest. Deforestation can affect forest-dependent bat species, which may lose both foraging and roosting habitats (Ellerbrok et al. 2022, Reusch et al. 2023). In addition, roads may cause fragmentation because of barrier effects, particularly for low-flying forest specialist bats (Fensome and Mathews 2016).

### Local displacement

The avoidance of wind turbines by bats has been observed for various species and functional guilds, both on farmland (Barré et al. 2018, Leroux et al. 2022, Reusch et al. 2022) and at forested sites (Ellerbrok et al. 2023, 2024, Gaultier et al. 2023), in a range of ecological zones in Europe, including the boreal zone (Gaultier et al. 2023), the Atlantic zone (Barré et al. 2018, Leroux et al. 2022, Reusch et al. 2022), and the continental zone (Ellerbrok et al. 2023, Reusch et al. 2023, 2024). For example, the activity of bent-winged bats (*Miniopterus* sp.) and wattled bats (*Chalinolobus* sp.) was 20 times lower at multiturbine facilities in New Caledonia than at nearby control sites without turbines (Millon et al. 2018). In Europe, the activity of edge-space- and open-space-foraging bat ensembles declined by 54% and 20%, respectively, on farmland up to 1 kilometer from wind turbines (Barré et al. 2018). A similar decrease was observed for open-space- and narrow-space-foraging bats at wind turbines in forests (Ellerbrok et al. 2022, Gaultier et al. 2023, McKay et al. 2023, Reusch et al. 2023). The responses of individuals are highly variable, but the repulsion effect tends to increase with the number and size of turbines (table 1; Reusch et al. 2022, 2023). The lowered activity of bats in proximity to wind turbines is not explained by depletion of local populations from mortality by wind turbines, because avoidance has also been reported for species at low risk of collision, such as forest specialist bats (Ellerbrok et al. 2022). Instead, it is likely that the displacement of bats is caused by turbine-generated noise as forest specialist bats only showed avoidance of operating wind turbines at relatively high wind speeds (Ellerbrok et al. 2024). Avoidance may be a general behavioral response, although it has not been widely studied in other areas than Europe. Habitat loss by displacement may become a major conservation concern for bats with expansion of wind energy facilities.

## Current practice of avoidance–mitigation–compensation hierarchy in wind energy projects

In many countries, wind energy development follows the mitigation hierarchy (e.g., Rodrigues et al. 2015), which includes avoidance, mitigation, compensation, and offset (Arlidge et al. 2018). We summarize options for effective measures to protect bats at wind turbines and provide a brief assessment of current practice.

### Avoidance

To protect bats, wind turbines should not be located in areas that support high bat diversity or are otherwise priorities for conservation (table 2; Rodrigues et al. 2015, Neri et al. 2019). Surveys for bat species richness can be enhanced by using multiple methods and at appropriate temporal and spatial scales (Rodrigues et al. 2015). Preconstruction acoustic surveys do not necessarily correspond to postconstruction acoustic activity or mortality rates (Lintott et al. 2016, Solick et al. 2020), especially when conducted over short time periods and local spatial scales. Nonetheless, bat activity rates during operation strongly correlate with fatalities (Peterson et al. 2021), and it is logical to assume that sites with higher bat activity pose a greater risk to bats, although mortality rates cannot necessarily be predicted.

**Table 2.** Examples of general guidance on best practices for siting of wind turbine facilities to minimize impacts on bats.

**General guidance**	**Justification**	**Example solution**	**Source**
No wind turbines in old or highly structured forests	Forest habitats have high diversity of bats, particularly many endangered species	Wind turbines sited in open or agricultural areas with low diversity	Ellerbrok et al. 2022, Gaultier et al. 2023
At least 500 meters away from small bat colonies (at most 100 bats), and more than 5 kilometers away from large colonies, such as in caves (at least 100 bats)	Proximity to bat colonies or large roosts results in higher abundance of bats at wind turbines, which increases collision risk	In the Caribbean, hot caves with thousands of bats should qualify as key biodiversity areas and benefit from more than 10 kilometers buffer without wind turbines	Ladle et al. 2012, Reusch et al. 2023
At least 500 meters away from known foraging or commuting habitats	Foraging and commuting areas likely have increased bat activity, which may increase the number of fatalities.	Wind turbines sited away from hedgerows and forest edges.	Heim et al. 2018, Leroux et al. 2022
Limited wind turbine density at migratory corridors	Avoidance behavior of bats toward wind turbines may impair migratory movements and may add energetic costs when bats fly around multiturbine facilities, increased abundance of bats along migratory corridors may increase the number of casualties at wind turbines	Wind turbine density should be limited in the area of migratory corridors, such as along the coastlines of the Baltic and North Sea in Europe	Rydell et al. 2014, Ijäs et al. 2017, Bach et al. 2022, Reusch et al. 2022

*Note:* We recognize local situations vary and that guidance from this table should be adjusted to reflect local conditions.

Regional biodiversity hotspots should be prioritized for conservation, and wind turbines should be sited at least 500 meters away from such areas (table 2). Outside these biodiversity hotspots, key bat habitats, such as wetlands and water bodies, should also be avoided (Mas et al. 2021). Forests and woodland edges are similarly important for many bat species (Russo et al. 2010, Meyer et al. 2016) and should not be used for wind energy production if alternatives are available (table 2; Rodrigues et al. 2015). In the absence of alternative sites in open landscapes, wind turbines should only be sited in forest plantations and forest habitats with low bat species richness and of overall low conservation value (Ellerbrok et al. 2022). Wind turbines should not be placed close to roosts with even small colonies of vulnerable bat species, specifically those used during pregnancy, lactation, mating, or hibernation (Reusch et al. 2023). For large colonies, greater offset distances may be needed (e.g., at least 5 kilometers for caves with colonies of several thousand individuals; table 2). As a precautionary approach, turbines should also not be placed close to swarming sites for bats.

Migration and commuting corridors are important landscape features for bats. Often, such corridors follow prominent landscape features (Wieringa et al. 2021), such as river valleys (Furmankiewicz and Kurcharska 2009) and coastlines (Hutterer et al. 2005, Voigt et al. 2018, Bach et al. 2022), some of which are also heavily used for wind energy production (Nunalee and Basu 2014, Wieringa et al. 2021). High densities of wind turbines in such corridors may affect bat habitat connectivity if bats are displaced by turbine operation (Reusch et al. 2022, Gaultier et al. 2023).

As for an evaluation of current avoidance practice, the current siting practices suggest that, although numerous tools and guidelines for bat-friendly siting of wind turbines are available (e.g., USFWS 2012, Rodrigues et al. 2015, Hise et al. 2022), the appropriate methods or approaches are largely ignored, even when regulations are legally binding (e.g., Barré et al. 2022, Voigt et al. 2022a). Although there are opportunities to potentially reduce bat mortality at wind turbines through sensitive siting, sites are often selected according to other criteria, such as access to transmission lines, local regulations (e.g., setback, flicker, noise), and land rental rates. However, optimal siting often does not reduce mortality rates to near zero and may have a limited effect on species attracted to turbines. For example, high fatality rates can still occur at wind facilities sited in open agricultural areas (Mathews et al. 2016, AWWI 2020, Scholz et al. 2023). Siting guidance is often based on the best available information, which can be limited in many areas where wind energy is expanding. In conclusion, optimal siting is important to protect key bat habitats, but siting alone will not reduce fatality rates to near zero.

### Mitigation

An effective way to reduce bat fatalities is to feather the blades below the cut-in speed, which reduces the rotational speed (Arnett et al. 2011). Blade feathering can reduce fatalities by about one-third, with negligible loss of electricity generation (Whitby et al. 2021). In addition, increasing the wind speed at which turbines begin generating electricity, a mitigation measure known as *curtailment*, can significantly reduce bat fatalities (Arnett et al. 2011, Brinkmann et al. 2011, Behr et al. 2017, Martin et al. 2017, Măntoiu et al. 2020, Smallwood and Bell 2020, Whitby et al. 2021, Bennett et al. 2022, Rabie et al. 2022, Barré et al. 2023). A recent review indicates that an increase of 1.5 meters per second in cut-in speed results in an average reduction in fatalities of 40%. Shifts in cut-in speed of more than 2.3 meters per second may lower fatalities by more than 63% (Whitby et al. 2021). Additional variables, such as ambient temperature, can be used to refine curtailment algorithms (box 2). For wind turbines in the temperate zone, the energy loss from curtailment schemes is approximately 1%–4% of a turbine's annual energy production (Arnett et al. 2011, Behr et al. 2017, Whitby et al. 2021), depending on the size and type of wind turbine, the local habitat, and the local bat activity. Sophisticated curtailment schemes can integrate information on bat acoustic activity, estimated mortality rates, and environmental and temporal variables (box 2). With the rapid expansion of wind turbines around the world, effective mitigation measures that reduce the risk of mortality to near zero are key to protecting bats.

Box 2. **Establishing curtailment criteria.**

**Figure 4.**
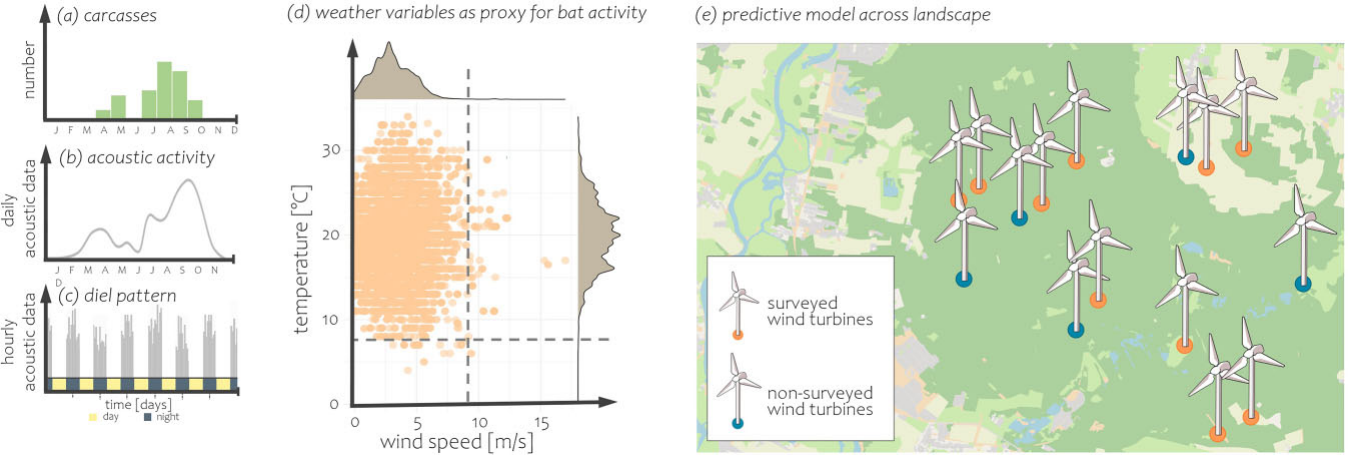
Schematic illustration of curtailment criteria (carcasses, acoustic activity, diel patterns, weather variables) and predictive models across landscapes.

Curtailment can be used to reduce bat mortality at wind turbines. Curtailment criteria have generally been implemented on the basis of carcass searches (figure 4a) and acoustic surveys at the turbine nacelle (figure 4b), which reveal seasonal (figure 4b) and diel (figure 4c) patterns of bat activity. The acoustic activity of bats at the nacelle, shown as orange circles in figure 4d in relation to ambient temperature and wind speed, can be used to refine threshold criteria for curtailing wind turbine operation on the basis of weather parameters (figure 4d)—for example, operating wind turbines only above a critical wind speed and below a critical ambient temperature. Blanket curtailment involves feathering the blades and limiting wind turbine operation during nights with low wind speeds, typically in late summer or fall. Blanket curtailment can reduce mortality by more than 80% (Măntoiu et al. 2020, Whitby et al. 2021, Bennett et al. 2022), although reductions are typically more modest (Adams et al. 2021).

Predictive approaches relate weather variables to the acoustic presence of bats in the first year or two after construction, so that these variables, rather than direct measures of bat fatalities, can be used for the remainder of turbine operation to guide curtailment (Behr et al. 2017). Importantly, the relationship between acoustic activity and mortality rates must be established in advance at reference turbines to derive an optimal curtailment algorithm for wind turbines on the basis of acoustic data alone (Behr et al. 2017). Alternatively, predictive landscape models use the relationship between fatalities and bat acoustic activity at the nacelle in a set of reference turbines to predict periods of high activity—and, therefore, fatality risk—at other wind turbine sites (figure 4e; Barré et al. 2023). This approach can be used in conjunction with habitat suitability models to identify sites with the lowest risk of mortality (e.g., distance from key habitats; Rodrigues et al. 2015). Predictive approaches have the potential to significantly reduce bat mortality while optimizing energy yield but are sensitive to violations of underlying assumptions. For example, these models assume that bats interact similarly with all types of turbines, that the relative coverage of acoustic monitoring and the variation in spatial distribution of bats are similar across regions and turbine types (Voigt et al. 2021, 2022b), and that the risk of fatality is similar within a given region because bat assemblages, turbine types, and weather conditions are assumed to be comparable. Often, the effectiveness of these mitigation measures, such as carcass searches, is not tested in the field.

Context-dependent approaches aim to manage turbine operations in real time, on the basis of the actual presence of bats (Hayes et al. 2019). Context dependent approaches rely on continuous acoustic monitoring or imaging, but it is rarely possible to achieve complete coverage of the rotor swept zone. Context-dependent approaches require continuous acoustic or visual monitoring and lack detailed validation, so such approaches have not been implemented on a larger scale, although technologies to enable such an approach are currently under investigation.

Acoustic deterrence, the intentional use of sound to mask bat echolocation, has been proposed as an effective way to reduce bat fatalities. Current evidence is equivocal, and validations of the effectiveness of acoustic deterrence remain lacking (e.g., Romano et al. 2019, Weaver et al. 2020, Gilmour et al. 2021, Good et al. 2022).

On a global scale, blade feathering and curtailment is practiced in only a few countries, such as parts of the United States, Canada, and sometimes in the European Union, where bats are legally protected. However, even within the European Union, the implementation of mitigation measures is often not considered. For example, more than 18,000 of the 30,000 onshore wind turbines currently installed in Germany operate without curtailment, even though some of these turbines kill large numbers of bats (Voigt et al. 2022a, Scholz et al. 2023). Despite the progress made in recent years in improving curtailment regimes, it is difficult to eliminate bat casualties at turbines completely (Behr et al. 2017, Hayes et al. 2019, Whitby et al. 2021). In areas with high wind turbine densities, cumulative mortality may cause population declines even when curtailment is consistently implemented. Although the current curtailment practices suggest that annual energy losses are small, this assumption may change for wind turbines operating in regions with year-round bat activity. Furthermore, in countries where there is no more space for wind turbines in high wind resource areas, they are increasingly being sited in lower wind resource areas, where the energy yield impact of curtailment could be greater.

### Compensation

The implementation of offset measures to compensate for impacts that cannot be avoided or reduced is a legal requirement in a few countries worldwide, such as the European Union (McGillivray 2012). Offsets are intended to provide population resiliency by restoring or creating habitat for species at high risk of collision (Regnery et al. 2013). Several approaches have been proposed as compensation measures, such as conversion of forest plantations into more diverse forests, improvement of the quality of existing roosts, provision of new roost structures, or creation of new water bodies as foraging habitats for bats (Peste et al. 2015). To our knowledge, the effectiveness of most of these proposed measures has not been validated (Berthinussen et al. 2021).

In general, several studies highlight the limitations of offsetting approaches to achieve no net loss of biodiversity (Regnery et al. 2013, Bezombes et al. 2019). Currently, there is a lack of evidence on effective mitigation measures for wind turbine casualties and habitat loss. Compensating for the losses of individuals caused by wind turbines is likely to be difficult, given the low fecundity of bats (Barclay and Harder 2003, Barclay et al. 2011). Therefore, avoidance and mitigation approaches are critical to minimize the wind energy–bat conflict.

## Enforcement of conservation actions

Conservation measures that are already available must be applied more consistently in practice. Several interest groups have a central role to play here. The implementation of nature conservation measures could be enforced using various approaches.

### Legal framework and international treaties

Globally, measures to protect bats from the impacts of wind energy developments are mostly implemented in countries with strong legal protections for bats. In the United States, this only happens for species listed under the Endangered Species Act, which generally occurs after populations have already declined to levels at risk of extinction (Henson et al. 2018). In European countries that are signatories to the Bern Convention or implement the EU Habitats Directive, bats are legally protected, regardless of population status (Rodrigues et al. Rodrigues et al. 2015). In South Africa, conservation measures are included in environmental approvals for projects (e.g., MacEwan et al. 2020). In the absence of a legal framework, the only way to protect bats is through voluntary consideration by wind energy project developers or through conditions imposed by financial institutions. Voluntary compliance by countries to protect vulnerable bat species at wind turbines is problematic on a global scale and therefore virtually nonexistent. Many bat species are migratory and therefore covered by the United Nations Environment Programme's (UNEP) Convention on the Conservation of Migratory Species of Wild Animals, which applies to 131 member states. However, even in Europe, where this UNEP program has been focused specifically on bats as part of the EUROBATS agreement (signed in Bonn in 1979 and in London in 1981), it has not proven effective in resolving the conflict between wind energy production and bat conservation (Barré et al. 2023). Such international agreements may benefit from stronger enforcement if more countries join the initiative—that is, EUROBATS or if countries on other continents create similar initiatives under the UNEP umbrella.

### Wind energy companies and associated stakeholders

Wind energy companies could impose biodiversity conservation measures on themselves as they develop wind turbine facilities around the world. However, this practice may be the exception rather than the rule, because the free market does not reward the higher costs associated with biodiversity conservation. Some members of the wind energy industry and related stakeholders appear to view regulations related to conservation measures as a barrier. For example, the Global Wind Energy Council reports do not contextualize their call for increased wind energy production to achieve net zero emissions by 2050 with the need to also conserve biodiversity—for example, by recognizing the Intergovernmental Science-Policy Platform on Biodiversity and Ecosystem Services. In particular, the negative environmental impacts of wind energy development; for example, on bat and raptor populations, are largely ignored, or the countermeasures are criticized as obstacles (GWEC 2023).

### Governments and lenders

Governments and lending institutions such as the World Bank Group, the International Finance Corporation, the European Bank for Reconstruction and Development, the Asian Development Bank, and the German Kreditanstalt für Wiederaufbau can insist on conservation standards as a condition for financing or subsidizing wind energy projects. To this end, postconstruction monitoring of wind energy projects should be made mandatory by lenders and the results made available to the public. As a first step, a consortium of lending institutions has recently published guidelines for postconstruction mortality monitoring according to good international industry practice to guide wind project development in emerging markets (IFC 2023). However, counting carcasses is not in itself a conservation measure and does not ensure that a development is environmentally sustainable. Lending institutions and governments should therefore enforce the implementation of conservation measures according to the mitigation hierarchy, in compliance with international agreements such as the Bern Convention (Diaz 2010) or the Convention on the Conservation of Migratory Species of Wild Animals. International standards and criteria for siting and operating conditions would help to level the playing field for wind companies in assessing the feasibility and viability of wind energy developments around the world.

## Emerging challenges to reconcile bat conservation with wind energy production

In recent years, research has developed effective measures to reduce the impact of wind energy production on bats, but the rapid development of the wind energy industry poses new challenges for both research and nature conservation.

### Geographic expansion of wind energy production

Wind energy production is expanding rapidly in emerging countries. For example, the annual increase in wind power is 4.1 GW for Brazil in 2022 (GWEC 2023), a global biodiversity hotspot (Myers et al. 2000, Mittermeier et al. 2011, Alves et al. 2018). Unfortunately, many countries, particularly those with a high bat species richness, lack basic information on which bat species are at risk for many (e.g., Bernard et al. 2014, Alves et al. 2018), and almost nothing is known of the risks to nonecholocating species (Valença and Bernard 2015). Assessment of the impacts on local bat assemblages is also hindered by a lack of basic information on population dynamics, and it is unclear whether mitigation measures, such as curtailment, are as effective in reducing bat mortality in these emerging markets as they are in temperate regions. In regions without strong seasonal changes in bat activity, curtailment would be necessary throughout the year, which may reduce the tolerance of wind energy companies and other stakeholders for such measures.

### Wind turbine technology

Wind turbines are getting larger because the energy yield is better for taller turbines with longer blades (e.g., Voigt et al. 2015). Modern wind turbines are also more efficient at low wind speeds, so that locations that were previously considered unprofitable are now suitable for wind energy generation. The trend for taller turbines may also result in greater overlap of the rotor swept area with the flight path of migrating bats (Roeleke et al. 2016, Roemer et al. 2017, Wellig et al. 2018, O'Mara et al. 2019, McCracken et al. 2021, Reusch et al.^ 2022^, ^2023^). In addition, the curtailment of larger wind turbines causes greater losses for companies per wind turbine than for small turbines at the same cut-in speed (Voigt et al. 2015). This could be an incentive for wind energy companies to challenge curtailment rules in court. Some modern turbines also operate with less ground clearance, the distance between the ground and the lowest point of the moving blade. This could affect species with a low flight height. In addition, carcass searches are more expensive, and estimates have greater uncertainty at large wind turbines because the area to be searched increases with the square of the blade length and because more distant areas may be covered by more vegetation than the graveled area in the immediate vicinity of the tower. Larger turbines may also limit the ability to efficiently survey bats using ultrasonic detectors because of strong geometric and atmospheric attenuation of high-frequency echolocation calls (Voigt et al. 2021). In addition, variations in the spatial distribution of bats may affect the ability to accurately predict the presence of bats in the rotor swept zone on the basis of a subsample within the range of the ultrasonic detectors (Voigt et al. 2022b). Furthermore, acoustic deterrence of bats—if it is shown to be successful in independent studies (e.g., Romano et al. 2019, Weaver et al. 2020, Gilmour et al. 2021, Good et al. 2022)—may be less efficient at wind turbines with longer rotor blades, because atmospheric and geometric attenuation limits the range of the acoustic signal (Voigt et al. 2021).

### Siting of turbines in sensitive ecosystems

Wind turbines are often built in areas where power lines from the turbines can be easily connected to the transmission grid (Bennett et al. 2023), often regardless of whether ecologically sensitive habitats are affected. However, biodiversity criteria should be as important as infrastructure criteria in the siting of wind turbines. Bat-friendly siting of wind turbines may involve trade-offs for the protection of other animal groups or for the protection of ecologically valuable habitats that are less relevant to bats. Solving these wicked problems requires a high level of scientific evidence and the ability to reflect on the facets of the specific trade-off (Game et al. 2014).

### Lack of conservation practice

Although curtailment is effective in reducing casualties (e.g., Arnett et al. 2011, Whitby et al. 2021, Box 2), it has been difficult to implement without regulatory mandates. Even in countries with strict regulations, only a fraction of wind turbines operate with a curtailment system (Barré et al. 2022, Voigt et al. 2022a, Scholz et al. 2023). The American Wind Energy Association (now American Clean Power) announced a voluntary best management practice for the United States to feather blades below the manufacturer's cut-in speed, and this practice is also advocated by the new EUROBATs resolution 9.4 (2022), but no information is available on its implementation. In conclusion, we advocate that governments, regulators, and lenders establish regulatory tools to ensure that wind energy projects operate in an environmentally sustainable manner.

## Conclusions

Globally, the direct and indirect impacts of wind energy on bats pose a challenge to local bat populations and global bat diversity. The further development and global expansion of wind energy production should take bat conservation into account, especially because simple technical solutions and implementable management measures exist. The negative impacts of wind energy on bats vary between bat foraging guilds and also between global ecosystems.

The current evidence, mostly from the temperate zone, suggests that aerial insectivores and fruit-eating pteropodids may be most vulnerable to collisions with wind turbines, whereas bats foraging at low heights and in dense vegetation may lose foraging areas as their habitats are converted to wind energy production sites or as wind turbine operations displace sensitive species.

Avoiding areas known to be of high bat value is a critical first step in preventing bat losses. Another effective measure to further reduce bat losses at wind turbines is to restrict the operation of wind turbines at times of high bat activity. Positive validation of these measures in the temperate zone suggests that they are likely to be effective in emerging economies.

Mitigation measures may not reduce mortality to zero, suggesting that multiple and complementary approaches should be used to reduce the long-term impacts on bat populations, particularly in the context of the predicted rapid increase in wind turbine construction rates. Compensatory measures, such as the restoration and creation of roosting and foraging habitats, may be a useful component of strategies to manage the impacts of wind turbines but are unlikely to be sufficient on their own to fully offset direct mortality.

We therefore call for more research into effective compensation measures to offset, at least partially, bat mortality from wind turbines. Stakeholders need general guidelines for the protection of bats in wind energy developments around the world. These should include effective avoidance, mitigation, and compensation measures, illustrated by best practice examples. Regulatory requirements from governments are needed to ensure that conservation measures are implemented. International organizations such as the UNEP and the IUCN and financial institutions will have a key role to play in promoting environmentally sustainable wind energy projects, recognizing that development can only be considered sustainable if it addresses both the biodiversity and climate crises.

## Supplementary Material

biae023_Supplemental_File

## Author Biography

Christian C. Voigt (voigt@izw-berlin.de) and Carolin Scholz (scholz@izw-berlin.de) are affiliated with the Leibniz Institute for Zoo and Wildlife Research, in Berlin, Germany. Enrico Bernard (enricob2@gmail.com) is affiliated with the Laboratório de Ciência Aplicada a Conservação da Biodiversidade at the Universidade Federal de Pernambuco, in Recife, Brazil. Joe Chun-Chia Huang (ecojoe.huang@gmail.com) is affiliated with the Department of Life Science at the National Taiwan Normal University, in Taipei City, Taiwan. Winifred F. Frick (wfrick@batcon.org) and Michael Whitby (mwhitby@batcon.org) are affiliated with Bat Conservation International, in Austin, Texas, in the United States. Christian Kerbiriou (christian.kerbiriou@mnhn.fr) is affiliated with the Centre d'Ecologie et des Sciences de la Conservation at the Muséum national d'Histoire naturelle and the Centre National de la Recherche Scientifique at Sorbonne Université Station Marine, in Concarneau, France. Kate MacEwan (kmacewan@west-inc.com) is affiliated with Western EcoSystems Technology, in Cheyenne, Wyoming, in the United States. Fiona Mathews (f.mathews@sussex.ac.uk) is affiliated with the School of Life Sciences at the University of Sussex, in Falmer, England, in the United Kingdom. Armando Rodríguez-Durán (arodriguez@bayamon.inter.edu) is affiliated with the Universidad Interamericana, in Bayamón, Puerto Rico. Paul W. Webala (paul.webala@gmail.com) is affiliated with the Department of Forestry and Wildlife Management at Maasai Mara University, in Narok, Kenya. Justin Welbergen (J.Welbergen@westernsydney.edu.au) is affiliated with The Hawkesbury Institute for the Environment at Western Sydney University, in Richmond, Victoria, in Australia.
